# Association d'une sarcoïdose pulmonaire, hépatite virale B et leucémie aigue myéloblastique

**DOI:** 10.11604/pamj.2014.18.163.876

**Published:** 2014-06-19

**Authors:** Illias Tazi, Hatim Nafil, Lahoucine Mahmal

**Affiliations:** 1 Service d'Hématologie, Chu Mohamed Vi, Université Cadi Ayyad, Marrakech, Maroc

**Keywords:** Sarcoïdose, hépatite virale, leucémie, Sarcoidosis, viral hepatitis B, leukemia

## Aux editeurs du Journal Panafricain de Médecine

Le lien entre la pathologie granulomateuse et cancer est suspectée depuis de nombreuses années. Cette association a longtemps été considéré comme fortuite. La prévalence de la sarcoïdose est également augmentée au cours des hémopathies malignes, notamment la maladie de Hodgkin (14%) et les lymphomes malins non Hodgkiniens (4 à 7%), plus rarement au cours du myélome et des leucémies aigues [1]. Il parait de plus en plus probable que la survenue de sarcoïdose maladie soit un événement de causes variées, encore mal définies, associant une prédisposition génétique et une exposition à des facteurs environnementaux spécifiques.

Nous rapportons le cas d'une patiente de 41 ans, suivie pour sarcoïdose pulmonaire depuis 6 mois sous corticothérapie orale et qui présente de manière concomitante des ménométroragies, un syndrome anémique et syndrome infectieux sans syndrome tumoral. L'examen gynécologique retrouve un polype utérin. L'hémogramme objective une anémie normo chrome normocytaire a 6.3 g /dl, un chiffre de leucocytes normal avec une neutropénie a 500 /mm3 sans blastes et un taux de plaquettes normal. Le myélogramme a montré un envahissement blastique supérieur a 70%. L'immunophénotypage a trouvé des bastes de nature myéloblastique (LAM) et le caryotype était normal. La sérologie de l'hépatite B était positive, ainsi la patiente était mise sous traitement antiviral. Une chimiothérapie d'induction été démarrée associant cytosine arabinoside et daunorubicine (protocole AML-03), la patiente était en échec thérapeutique en fin d'induction (myélogramme à J15 d'induction: 50% de blastes), d'où la décision d'une intensification avec une réponse favorable en fin d'intensification (myélogramme d’évaluation<3% de blastes). Actuellement la malade est en rémission après quatre cures de chimiothérapie avec un recul d'un an.

Vu la rareté et l’étipathogénie incertaine de cette triple association, nous avons jugé qu'il était utile de rapporter ce cas. En effet, les granulomatoses systémiques de type sarcoïdose peuvent survenir avant, pendant ou après un cancer. Cette éventualité est rare mais les dernières études confirment l'existence d'un lien privilégié entre les deux pathologies notamment pour les hémopathies malignes, les cancers du testicule, les cancers broncho-pulmonaires, les mélanomes et les hépato carcinomes. L'existence d'une réaction sarcoïdosique semble être associée à un meilleur pronostic du cancer en particulier pour la maladie de Hodgkin et les adénocarcinomes de l'estomac; le processus granulomateux pourrait ainsi jouer un rôle de barrière (suffisant ou insuffisant) à la diffusion métastatique. Il est difficile d'affirmer que les trois entités sont liées d'une part on ne possède que peu de données sur l'association hépatite virale B (HVB) et sarcoïdose (la sarcoïdose est-elle déclenchée par un traitement anti-HVB ‘ ou est-elle due à une réactivation préexistante chez les patients HVB positif ‘) et d'une autre part le lien de causalité LAM et sarcoïdose est difficile à confirmer.

L'association sarcoïdose et hémopathies malignes n'est pas fortuite. Une sarcoïdose qui fait suite à une LAM est une situation extrêmement rare, uniquement quelques cas ont été publiés dans la littérature [2, 3]. Celle-ci peut précéder la LAM de 3 à 7 ans [4].

Nous ne savons pas si réellement la sarcoïdose peut prédisposer à une LAM. Reich [4] a supposé que la sarcoïdose peut représenter une réponse immunologique à un antigène tumoral. Dans les tissus atteints de sarcoïdose, les cellules T helper (CD4) positives de type Th1 sont augmentées en nombre et ont un phénotype activé. Ces lymphocytes CD4 expriment un récepteur T au répertoire oligoclonal suggérant un mécanisme de stimulation antigénique à la base de ce processus inflammatoire bien qu'aucun antigène cible n'ait été identifié jusqu’à ce jour [5]. Cette stimulation poly clonale B observée dans la sarcoïdose, particulièrement dans les formes chroniques de la maladie, pourrait conduire à l’émergence d'un clone malin et d'une maladie lympho-proliférative.

Un autre problème qu'il faut aborder, c'est que la présence de cette association doit toujours inciter le clinicien à confirmer la rechute tumorale par un prélèvement biopsique; à l'inverse, toute réaction granulomateuse ne peut être attribuée à une sarcoïdose qu'après avoir correctement éliminé toute cause secondaire notamment néoplasique.

Les réactions granulomateuses observées au cours de certaines néoplasies pourraient constituer, comme dans d'autres situations (infections, toxiques...), une réaction de défense de l'hôte vis-à-vis d'un antigène tumoral, permettant d'expliquer un pronostic parfois plus favorable en regard de certains cancers. Cette observation illustre les problèmes posés par l'association sarcoïdose, hépatite virale B et leucémie aigue myéloblastique: mode de révélation particulier, complication ou association fortuite mais trompeuse. Des études prospectives seraient utiles afin de définir certaines associations morbides significatives et préciser le type de recherche étiologique à réaliser après la découverte d'une sarcoïdose.
